# Driving frugal innovation in SMEs: how sustainable leadership, knowledge sources and information credibility make a difference

**DOI:** 10.3389/fsoc.2024.1344704

**Published:** 2024-03-01

**Authors:** Khalil Ur Rehman, Rana Salman Anwar, Valentin Marian Antohi, Uzma Ali, Costinela Fortea, Monica Laura Zlati

**Affiliations:** ^1^Department of Management Sciences, Khawaja Fareed University of Engineering and Information Technology, Rahim Yar Khan, Pakistan; ^2^Business Administration Department, Sukkur IBA University, Sukkur, Pakistan; ^3^Department of Business Administration, Dunarea de Jos University of Galati, Galati, Romania

**Keywords:** sustainable leadership, frugal innovation, sources of knowledge, information credibility, small and medium enterprises

## Abstract

This study investigates the driving factors behind frugal innovation in Small and Medium-sized Enterprises (SMEs). It specifically examines sustainable leadership as an independent variable, considering its impact on frugal innovation, with sources of knowledge mediating this relationship and information credibility moderating the effects. Employing a Partial Least Squares Structural Equation Modeling (PLS-SEM) approach, data were gathered from 325 employees of SMEs in Pakistan. This methodology was chosen for its ability to handle complex relationships between multiple variables simultaneously, offering robust insights into the interplay among sustainable leadership, sources of knowledge, information credibility, and frugal innovation. The results reveal significant associations between sustainable leadership, sources of knowledge, information credibility, and frugal innovation. Sustainable leadership demonstrates a substantial influence on both sources of knowledge and frugal innovation. Furthermore, sources of knowledge play a vital role in mediating the relationship between sustainable leadership and frugal innovation. Information credibility emerges as a significant moderator, affecting the pathways between sustainable leadership, sources of knowledge, and frugal innovation. The findings underscore the importance of sustainable leadership and credible information sources in driving frugal innovation within SMEs. They highlight the intricate interdependencies among these variables and emphasize the pivotal role of information credibility in shaping these dynamics. These results carry significant implications for SMEs in Pakistan, shedding light on the mechanisms through which sustainable leadership and reliable knowledge sources can stimulate frugal innovation in emerging economies.

## Introduction

The role of Small and Medium-sized Enterprises (SMEs) in the economic development of Pakistan has been underscored in recent studies (Khan et al., 2021; Manzoor et al., 2021). These enterprises significantly contribute to employment generation and play a pivotal role in fostering the country’s GDP growth (Jamal et al., 2021). However, the growth and competitiveness of SMEs are impeded by various challenges related to resources, finance, and innovation (Litvinenko and Sergeev, 2019). One specific obstacle identified is the absence of frugal innovation, characterized by the capacity to develop novel products, processes, or services with minimal resources and cost-effectiveness (Hyypiä and Khan, 2018).

In response to these challenges, frugal innovation has emerged as a promising strategy for SMEs seeking to overcome resource constraints and attain sustainable growth (Hyypiä and Khan, 2018). Noteworthy factors influencing frugal innovation in SMEs include sustainable leadership and knowledge sources (Iqbal and Piwowar-Sulej, 2023). Sustainable leadership is defined by the incorporation of economic, environmental, and social considerations into decision-making processes by leaders (Iqbal and Ahmad, 2021). Concurrently, knowledge sources refer to the avenues through which SMEs acquire the necessary knowledge and information to propel their innovation endeavors (Asada et al., 2020). This research aims to delve into the interplay of sustainable leadership and knowledge sources as critical drivers of frugal innovation within the context of SMEs in Pakistan.

Frugal innovation has emerged as a promising strategy for SMEs to overcome resource constraints and achieve sustainable growth. Sustainable leadership and knowledge sources are critical factors that drive frugal innovation in SMEs. Sustainable leadership refers to the ability of leaders to integrate economic, environmental, and social considerations into their decision-making process. Knowledge sources, on the other hand, refer to the channels through which SMEs acquire knowledge and information to drive their innovation efforts.

The primary objective of this research is to assess the influence of sustainable leadership and sources of knowledge on the implementation of frugal innovation within SMEs in Pakistan. Furthermore, the study seeks to investigate the moderating effect of information credibility (AlMulhim, 2021) on the association between sustainable leadership and frugal innovation, as well as between sustainable leadership and sources of knowledge. Additionally, this study examines the mediating role of sources of knowledge (Iqbal and Piwowar-Sulej, 2023) in the link between sustainable leadership and frugal innovation.

Recent studies have highlighted the importance of frugal innovation in SMEs, particularly in emerging economies like Pakistan (Subhan et al., 2013; Bhutto, 2021; Khattak et al., 2022). However, little attention has been given to the role of sustainable leadership and knowledge sources in driving frugal innovation (Lei et al., 2021; Dubey et al., 2022; Iqbal et al., 2022). This study aims to fill this gap in the literature by providing insights into the factors that drive frugal innovation in SMEs.

In light of the COVID-19 pandemic, SMEs in Pakistan face unprecedented challenges (Hossain et al., 2022). The pandemic has disrupted supply chains, decreased demand for goods and services, and forced SMEs to adopt new ways of working. In this context, frugal innovation has become even more critical for SMEs to survive and thrive (Broadbent et al., 2019; Yousaf et al., 2022). This study aims to provide valuable insights into the drivers of frugal innovation, which can help SMEs in Pakistan navigate the current crisis and build resilience for the future.

This study’s overarching goal is to learn more about how SMEs in Pakistan are utilizing sustainable leadership, knowledge sources, and cost-cutting or frugal innovation. The study’s overarching goal is to investigate how sustainable leadership influences frugal innovation and access to knowledge sources at Pakistan’s SMEs. The research also seeks to examine the connection between sustainable leadership and cost-cutting or frugal innovation in Pakistani Businesses, as well as the connection between sustainable leadership and the information creadibility. The research also hopes to determine whether or not knowledge sources influence the connection between sustainable leadership and frugal innovation among Pakistani Businesses. Lastly, this research intends to ascertain whether the credibility of information mediates the connection between sustainable leadership and frugal innovation in Businesses in Pakistan. This study’s overarching goal is to help Businesses and policymakers in Pakistan gain a better grasp of the forces that motivate cost-saving innovation.

This study has important implications for SMEs, policymakers, and academics. By understanding the factors that drive frugal innovation, SMEs can develop strategies to overcome resource constraints and achieve sustainable growth. Policymakers can use the findings of this study to design policies that support the development of frugal innovation in SMEs. Academics can use the results of this study to expand the literature on frugal innovation in emerging economies.

The following parts of this research work offer a thorough examination of how sustainable leadership and knowledge sources contribute to cost-effective or frugal innovation among Pakistan’s small and most dynamic businesses. Credibility of information as a moderator and knowledge sources as mediators in these associations will also be investigated. Lastly, the study will address what these results indicates for SMEs, government officials, and researchers.

## Literature review and hypotheses development

### Frugal innovation and sustainable leadership

Empirical studies exploring the dynamics of driving frugal innovation in Small and Medium-sized Enterprises (SMEs) have gained prominence, particularly within the context of sustainable leadership, knowledge sources, and information credibility (Broadbent et al., 2019; Yousaf et al., 2022). Research on sustainable leadership in SMEs has investigated how leaders integrate economic, environmental, and social considerations into decision-making processes, influencing the frugal innovation landscape. Scholars (Lei et al., 2021; Dubey et al., 2022; Iqbal et al., 2022) have examined the role of knowledge sources as channels through which SMEs acquire and leverage information to foster innovation. Additionally, the empirical literature has delved into the significance of information credibility in shaping the frugal innovation capabilities of SMEs (Abdulmuhsin et al. 2021). This body of research seeks to empirically elucidate the nuanced relationships between sustainable leadership, diverse knowledge sources, information credibility, and their collective impact on driving frugal innovation within the SME sector. Insights from these empirical studies contribute to a comprehensive understanding of the mechanisms that propel frugal innovation in SMEs, offering valuable implications for practitioners, policymakers, and scholars alike.

Frugal innovation refers to the practice of developing cost-effective solutions that address the needs of resource-constrained markets, often leveraging limited resources to create high-value products or services (Cuhadar and Rudnák, 2022). Sustainable leadership involves the ability of organizational leaders to navigate and drive businesses toward sustainable practices, balancing economic success with social and environmental responsibilities (Hossain et al., 2022). However, innovation is the key to organizational success and competitive advantage (Jotabá et al., 2022), which makes organizations focus on innovation management. Through this process, they emphasize generating new ideas that are valuable and growth-oriented for organizations (Santos et al., 2019). Recently, frugal innovation has gained traction in innovation management studies (Iqbal et al., 2022). It is a “resource scare solution” that focuses on producing cost-effective products (Le et al., 2022). It has shifted organizational focus toward achieving sustainability, resilience, affordability, quality, and effectiveness through frugal innovation (Farooq, 2017). Niroumand et al. (2020) provide some critical enables of frugal innovation, which includes organizational culture, human capital, knowledge, R&D strategies, marketing strategies, management support, and environmental aspect.

In retrospect, the studies on innovation have identified a strong link between leadership styles and organizational innovation (Haroon et al., 2019). The frugal innovation capability of a firm is determined by intellectual capital and leadership characteristics (Cuhadar and Rudnák, 2022). For instance, according to Le (2021), the transformational leadership style is a precursor for inducing frugal innovation within an organization. Likewise, research has emphasized sustainable leadership, which helps foster innovation by effectively dealing with environmental challenges (Xuecheng and Iqbal, 2022). Sustainable leadership promotes employees’ innovative behavior by inducing employee creative self-efficacy and encouraging participative behaviors (Uppathampracha and Liu, 2022). According to Iqbal et al. (2022), a significant positive relationship exists between sustainable leadership and frugal innovation. Given the supportive and positive impact of leadership on innovation, this study hypothesizes that:

*H1:* Sustainable leadership significantly impacts frugal innovation.

### Sources of knowledge and sustainable leadership

Le et al. (2022) emphasize that leadership plays a very crucial role in knowledge management, and good leadership facilitates knowledge flow in an organization (Al Amiri et al., 2020). However, an exploitative leadership style may halt the knowledge management process (Abdulmuhsin et al., 2021). There are two ways to increase one’s knowledge base: (1) within one’s own organization, through experiences and training; and (2) through external sources-exposure to information and ideas from outside one’s own organization (Hervas-Oliver et al., 2021). In a business setting, for instance, executives persuade, instruct, and inspire their employees (the firm’s internal knowledge sources) to learn and share what they have learned with others (Naqshbandi and Tabche, 2018). Similarly, sustainable leadership focuses on attaining triple goals of economy, society, and environment (Cuhadar and Rudnák, 2022). Sustainable leaders develop policies and design mechanisms that value the knowledge and experiences of people (Hargreaves, 2007), thus supporting knowledge sources. Given the supportive influence of leadership on sources of knowledge, this study hypothesizes that:

*H2:* Sustainable leadership significantly impacts the sources of knowledge.

### Sources of knowledge, sustainable leadership, and information credibility

Information plays a crucial role in people’s lives as they constantly seek information (Jamal et al., 2021). However, the credibility of the information matters for the information seekers. Literature defines credibility as the believability of a person or an object (Yin et al., 2018). Information credibility is one of the dimensions of information quality, representing people’s trust in the information they seek or receive (Tseng, 2022). Information credibility is an important aspect that helps make accurate evaluations of obtained information (Jamal et al., 2021). Naqshbandi and Jasimuddin (2018) argue that leadership has an impact on the credibility of various types of information. To this end, a study by Aby (2020) suggests that information credibility and sustainable leadership are interlinked. Also, literature considers a leader a source of information in an organization (Khan et al., 2017) because sustainable leaders are great knowledge influencers (Reid, 2014); they support knowledge creation and mobilization processes. Therefore, a positive leadership style strengthens knowledge acquisition in an organization (Al Amiri et al., 2020). At the same time, hostile or exploitative leadership styles inhibit the flow of information (Abdulmuhsin et al., 2021). Therefore, this study proposes that sustainable leadership as a positive style stimulates an informative environment within an organization, which affects the sources of knowledge where information credibility plays an essential role in strengthening or weakening this relationship.

*H3:* Information credibility plays a moderating role in the path of sustainable leadership and frugal innovation.

*H4:* Information credibility plays a moderating role in the path of sustainable leadership and sources of knowledge.

### Frugal innovation and sources of knowledge

The extant body of literature provides enough evidence of the essential role of knowledge in harnessing innovation (Salunke et al., 2019). However, this evidence is still limited on which kind, quality, and knowledge sources best support frugal innovation (Lei et al., 2021). Frugal innovation is unique from other types of innovation as it focuses on a triple-bottom-line strategy—social effect, environmental advantages, and commercial prospects (Liao, 2022). Achieving such results necessitate specialized knowledge from multiple sources (Hervas-Oliver et al., 2021). Frugal innovation is aided by multiple knowledge sources, as within an organization. The knowledge enters through internal and/or external sources (Shehzad et al., 2022). According to Dost et al. (2019), frugal innovation is the combined result of internal knowledge developed by the company and external knowledge sourced from outside. According to Shehzad et al. (2022), internal and external knowledge sources help organizations acquire frugal innovation. Given the supportive influence of sources of knowledge on innovation, this study hypothesizes that:

*H5:* Sources of knowledge significantly impact frugal innovation.

### Frugal innovation, sustainable leadership, and the role of sources of knowledge

Al-Husseini et al. (2021) state that leadership, knowledge, and innovation are interrelated. Knowledge is the core of any innovation, specifically when the innovation is frugal (Shehzad et al., 2022). Knowledge sources enable the transfer of sensitive and specific information (Abdullah et al., 2020). They are crucial for value creation through reintegrating businesses, which helps in focusing on product quality, cost-effectiveness, and innovation. According to Svetina and Prodan (2008), knowledge sources impact a firm’s innovative capability and processes. External sources of knowledge such as national, international, and internal sources such as a firm’s R&D, continuous improvement, employee training, and in-house knowledge help bring innovation. For frugal innovation, effective knowledge management accelerates the overall organizational innovation process (Dost et al., 2019). Likewise, leadership influences sources of knowledge. Leaders who desire control over subordinates negatively influence knowledge sources as they create a risk-aversive and conservative workplace environment (Serrano-Bedia et al., 2016). Frugal innovation is derived through knowledge generation, and sustainable leadership impacts knowledge generation by encouraging employees’ risk-taking behaviors (Iqbal et al., 2022; Shehzad et al., 2022). Hence, this study proposes that sustainable leadership creates a positive influence on knowledge sources which in turn promotes frugal innovation; therefore, it hypothesizes that:

*H6:* Sources of knowledge significantly mediate the relationship between sustainable leadership and frugal innovation.

### Sources of knowledge, sustainable leadership, and the role of information credibility

Leadership remains one of the critical factors impacting the sources of knowledge (Hargreaves, 2007; Cuhadar and Rudnák, 2022), whereas its mediation role in linking leadership with innovation is also evident in the present body of literature. Abro et al. (2020) report that the credibility of the knowledge source is determined by the credibility of the information. Likewise, the information acquired from credible sources helps leaders and their followers make effective decisions (Abro et al., 2020; Hallo et al., 2020). The study by Aby (2020) also reckons that leadership and information credibility correlate with each other; also, Khan et al. (2017) suggest that leaders are the source of (credible) information in an organization. Domingos et al. (2022) also suggest that leaders evaluate the credibility of sources by thoroughly analyzing the quality of information. However, limited evidence is available in the literature on the moderating role of information credibility. Besides, AlMulhim (2021) also suggested using information credibility as moderation on the path of frugal innovation. Henceforth, given the earlier moderation of information credibility on the path of sustainable leadership and sources of knowledge, this study also hypothesizes that information credibility equally moderates the mediated path of sources of knowledge in the relationship between sustainable leadership and frugal innovation. The developed hypothesis is as follows:

*H7:* In the mediating role of sources of knowledge in the relationship between sustainable leadership and frugal innovation, information credibility plays a significant moderating role on the path of sustainable leadership and sources of knowledge.

## Methodology

Taking into consideration the moderating effect of information credibility and the mediating effect of knowledge sources, this study’s main goal was to examine the impact of sustainable leadership on frugal innovation through Businesses in Pakistan. This study used a cross-sectional design so data was gathered through the use of a quantitative research technique and surveys. Data analysis was performed using PLS-SEM. The targted respondents of this research were the employees of SMEs in Pakistan who were involved in the production of services or goods. The inclusion criteria were as follows: (i) the participants should be currently employed in an SME, (ii) they should have a minimum of 1 year of work experience, and (iii) they must be willing to take part in the research. Participants who were recruited for the research but did not fulfil the eligibility requirements were not included. Additionally, seven incomplete questionnaires and responses with missing data were also excluded from the analysis.

### Measurement

The information was gathered using a survey questionnaire. The questionnaire consisted of five sections: (i) demographic information, (ii) sustainable leadership, (iii) sources of knowledge, (iv) information creadibility and (v) frugal innovation. The study used the following scales for data collection: (i) a ten-item scale of sustainable leadership by Dalati et al. (2017), (ii) a ten-item scale of frugal innovation by Khattak et al. (2022), (iii) a six-item scale of sources of knowledge by Broadbent et al. (2019), and (iv) a four-item information credibility scale developed by Li and Suh (2015).

The correlations between the factors were analyzed using PLS-SEM 4.0 for this research. In this research, bootstrapping with 5,000 repetitions was used to estimate the importance of the path coefficients. Ethical standards for studies involving human participants were followed throughout this study. Participants were told what the goals of the study were and that they could stop taking part at any time. No personal information was asked of the participants, so their privacy and anonymity were kept. In conclusion, a quantitative research strategy was used to gather information from 325 workers at Pakistani SMEs. The study utilized PLS-SEM for data analysis and selected appropriate scales for data collection. The study followed ethical guidelines and included and excluded participants based on the inclusion and exclusion criteria.

### Statistical procedure

In this study, PLS-SEM was employed to analyze the data and examine the relationships between the variables (Ringle et al., 2015). In order to determine the spread, shape, and bias of the population sampling distribution, the Bootstrapping technique was utilized for 5,000 iterations (Hair et al., 2019). Traditional covariance-based SEM (CBSEM) was chosen over PLS because it could not handle multivariate normality, model complexity, measurement level, sample size, and uncertainty (Hair et al., 2019). This study’s primary goal was to construct and assess a theoretical model.

Cronbach’s alpha analysis was used to figure out how consistent the variables were among themselves. The numbers confirmed that the measures used to assess the variables are very consistent with each other. The Cronbach’s alpha for the dependent variable, “Frugal Innovation, “was 0.947. This means that the measurement of this variable is very reliable. The alpha coefficient for Information Credibility is 0.879, suggesting a high level of consistency in the measurement of this moderating variable.

The mediating variable, “Sources of Knowledge, “has a Cronbach’s alpha of 0.939, which means that the way this variable is measured is very consistent. Lastly, the independent variable, Sustainable Leadership, has an alpha coefficient of 0.962, which means that measurement of this variable is very reliable. Cronbach’s alpha values from this study are higher than the recommended minimal level of 0.7 for research in the social sciences. This means that the measures employed for each variable are reliable and have adequate internal consistency for measuring the concepts they represent. These results show that the questionnaire used to get information from the participants is a good way to measure the study’s variables. All variables have a high level of internal consistency, which indicates that the individuals who took part in the study understood the questions and gave consistent answers. This makes the study more reliable. In conclusion, the results of Cronbach’s alpha analysis show that the measures used for frugal innovation, information credibility, sources of knowledge, and sustainable leadership are reliable and consistent with each other; as the values of them are in acceptable range (Tavakol and Dennick, 2011). This shows that these scales can be used for further analysis (see Table 1).

**Table 1:** Cronbach’s alpha values for variables.

	Cronbach’s Alpha
Frugal innovation	0.947
Information credibility	0.879
Sources of knowledge	0.939
Sustainable leadership	0.962

## Statistical analyses

In Table 2, the average variance extracted (AVE), factor loadings, and composite reliability values for the four variables are shown. All indicators for each variable exhibited factor loadings in excess of the 0.50 criterion, demonstrating high levels of construct validity. The composite reliability values for all variables ranged between 0.917 and 0.967, indicating the superior internal consistency and reliability of the measurement scales used in this study.

**Table 2:** Loadings, composite reliability, and average variance extracted.

	Item	Loading	Composite reliability	Average variance extracted
Frugal innovation	FI- 1	0.832	0.954	0.676
	FI- 10	0.821		
	FI- 2	0.785		
	FI- 3	0.835		
	FI- 4	0.843		
	FI- 5	0.839		
	FI- 6	0.803		
	FI- 7	0.811		
	FI- 8	0.841		
	FI- 9	0.808		
Information credibility	IC- 1	0.817	0.917	0.734
	IC- 2	0.848		
	IC- 3	0.876		
	IC- 4	0.883		
Sources of knowledge	SK- 1	0.892	0.952	0.768
	SK- 2	0.910		
	SK- 3	0.877		
	SK- 4	0.844		
	SK- 5	0.889		
	SK- 6	0.844		
Sustainable leadership	SL- 1	0.854	0.967	0.744
	SL- 10	0.857		
	SL- 2	0.856		
	SL- 3	0.837		
	SL- 4	0.822		
	SL- 5	0.890		
	SL- 6	0.869		
	SL- 7	0.877		
Student engagement	SL- 8	0.882		
	SL- 9	0.881		

In addition, the AVE values were above the cutoff value of 0.50, which suggests that the variables under study were accurately assessed and possessed high convergent validity. The AVE values for Frugal Innovation, Information Credibility, Knowledge Sources, and Sustainable Leadership ranged from 0.676 to 0.768.

The indicators for each variable in this study exhibited factor loadings above the recommended threshold of 0.50, indicating good levels of construct validity. These results suggest that the measurement scales used in this study are reliable and valid, thus providing a solid foundation for data analysis and interpretation. The results show that the four variables studied have high internal consistency, reliability, and convergent validity. This means that the measurement scales can be taken into account reliable and valid for future research in this field.

## Discriminant validity in structural equation modeling

Table 3 shows the results of the Fornell-Larcker Score, which was used to test the discriminant validity of the four factors that were looked at. The Fornell-Larcker Criterion compares the square root of each variable’s extracted average variance (AVE) to its correlation coefficient across all other variables. As shown in the preceding matrix (Table 3), the AVE values for each of the four variables were higher than their correlation coefficients. This shows that the variables can be used for further analysis. For instance, the AVE value for Frugal Innovation (FI) was 0.676, while its correlation with Information Credibility (IC), Sources of Knowledge (SK), and Sustainable Leadership (SL) was 0.822, 0.831, and 0.775, respectively. These results suggest that the constructs in this study are distinct and measure different concepts, and therefore, the study’s findings can be interpreted with confidence. Since the AVE for FI is higher than its correlation with the other variables, it can be concluded that FI has good discriminant validity.

**Table 3:** Fornell-Larcker criterion.

	FI	IC	SK	SL
Frugal innovation (FI)	0.822			
Information credibility (IC)	0.902	0.857		
Sources of knowledge (SK)	0.931	0.876	0.876	
Sustainable leadership (SL)	0.921	0.847	0.882	0.863

Similarly, the AVE for Information Credibility (IC) is 0.734, while its correlation with SK and SL is 0.876 and 0.847, respectively. The AVE for SK is 0.768, while its correlation with SL is 0.882. The AVE for SL is 0.744, indicating good discriminant validity for all four variables in the study. Overall, the results of the Fornell-Larcker Criterion suggest that the four variables in this study have good discriminant validity, meaning they are measuring distinct constructs and not overlapping with each other (Table 4).

**Table 4:** Effect size.

	FI	IC	SK	SL
Frugal innovation				
Information credibility	0.140		0.095	2.194
Sources of knowledge	0.240			0.787
Sustainable leadership	0.168		0.176	

### Inner model analysis

The present research paper utilized ‘PLS-SEM’ to construct the relationship model. SmartPLS 4.0 was employed for path analysis, which focused on effect size (*f*^2^) and predictive ability (*Q*^2^), as well as *R*^2^, beta (*β*), and *t*-value, following the guidelines of Hair et al. (2017). The R-square values for the endogenous variables, Frugal Innovation and Sources of Knowledge, were calculated and presented in Table 5. The R-square value for Frugal Innovation was 0.944, which means that the independent and mediating variables included in the model explained 94.4% of the variance in Frugal InnovationThe R-square value of Sources of Knowledge was 0.874, implying that 87.4% of the variance in Sources of Knowledge was explained by the independent variable Sustainable Leadership.

**Table 5:** *R*^2^ values of variables.

	R Square
Frugal innovation	0.944
Sources of knowledge	0.874

These results suggest that the model explains a large proportion of the variance in both Frugal Innovation and Sources of Knowledge. This indicates that the model is well specified and provides a good fit to the data. However, it is important to note that there may be other factors that contribute to Frugal Innovation and Sources of Knowledge that are not included in the model. Future studies researchers could explore additional variables to better explore the factors that influence Frugal Innovation and Sources of Knowledge in SMEs in Pakistan.

The Table 6 presents correlations among frugal innovation, information credibility, sources of knowledge, and sustainable leadership. Each cell indicates the degree of association between the corresponding factors. Notably, there’s a positive correlation between ic and SK (2.194), indicating a strong relationship. Additionally, moderate positive correlations are observed between FI and IC (0.140), SK and FI (0.240), SK and SL (0.787), and SL and IC (0.176). These insights shed light on the interconnected dynamics of these factors within the realm of innovation and sustainable leadership.

**Table 6:** Model fit predictive relevance of model.

	Saturated model	Estimated model	SSO	SSE	Q^2^ (=1-SSE/SSO)
SRMR	0.040	0.049			
d_ULS	0.762	8.795			
d_G	0.702	2.118			
Chi-square	1220.391	2853.799			
NFI	0.884	0.929			
Frugal innovation			3250.000	1236.996	0.619
Sources of knowledge			1950.000	694.115	0.644

In addition, it is essential to account for further analyses and theoretical considerations when interpreting these outcomes. Before testing the hypotheses, the researchers evaluated the Variance Inflation Factor (VIF), which ranged from 1.000 to 4.201, all below 5. This suggests that the predictor latent variables were not excessively similar, which was a concern highlighted by Hair et al. (2017).

Several measures, shown in Table 6, were used to judge how well the model fit the research. The Saturated Model showed a better fit than the Estimated Model, as indicated by the lower values of SRMR, d_ULS, and d_G, and higher values of NFI. The Chi-Square value was significant, indicating that the Estimated Model did not perfectly fit with the data. The SSO for Frugal Innovation was 3250.000, the SSE was 1236.996, and the Q^2^ was 0.619, showing that 61.9% of the variation in Frugal Innovation was explained by the model. In the same way, the SSO was 1950.000, the SSE was 694.115, and the Q^2^ was 0.644 for the mediating variable Sources of Knowledge. This means that the model explained 64.4% of the variation in Sources of Knowledge.

The provided table presents the results and interpretation of the model fitness of the research study. In the first column, the saturated model is shown. In the second column, the estimated model is shown. The third column displays the Sum of Squares due to the Model (SSO), and the fourth column displays the Sum of Squares due to Errors (SSE). Last, Q2 is shown in the fifth column. It shows how much of the variation in the dependent variable can be explained by the independent variable. As a measure of how well the model fits, the Standardized Root Mean Residual (SRMR) is used. Lower values show a better fit. The SRMR for the saturated model was 0.040, while the SRMR for the estimated model was 0.049. This shows that the model fits well.

This research study employed various measures to evaluate the fitness of the model, including d_ULS and d_G, which indicate model fit and are considered good when their values are between 0 and 1, with lower values indicating better fit. The obtained d_ULS and d_G values in this study were 0.762 and 0.702, respectively, indicating acceptable model fit.

**Figure 1:**
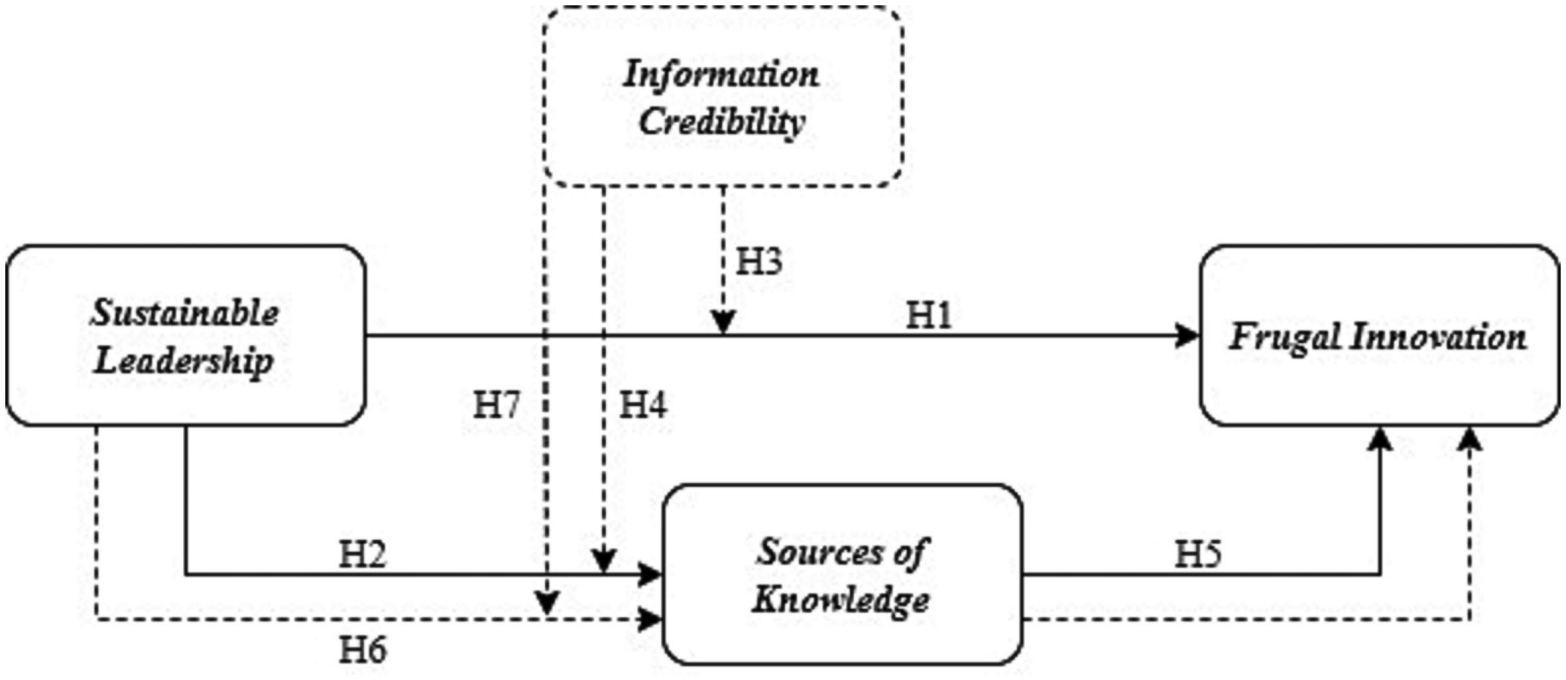
Conceptual model.

Additionally, this study’s Chi-Square value, which gauges the disparity between observed and model correlation matrices were substantially indicating an average model fit. The NFI was also used to figure out how well the model fit was. Values between 0 and 1 mean that the model fits well. The NFI values for both the saturated and estimated models were 0.884 and 0.929, which indicates an acceptable model fit. Lastly, the Q^2^ values for the dependent variables, Frugal Innovation and Sources of Knowledge, were 0.619 and 0.644, respectively. These values indicate that 61.9 and 64.4% of the variance in Frugal Innovation and Sources of Knowledge, respectively, were explained by the independent variables (Figure 1).

Overall, the results suggest acceptable model fit, with some areas for improvement, such as the significant Chi-Square value. Future studies may benefit from addressing these limitations to further enhance the model’s fit.

### Structural model path analysis results

**Figure 2:**
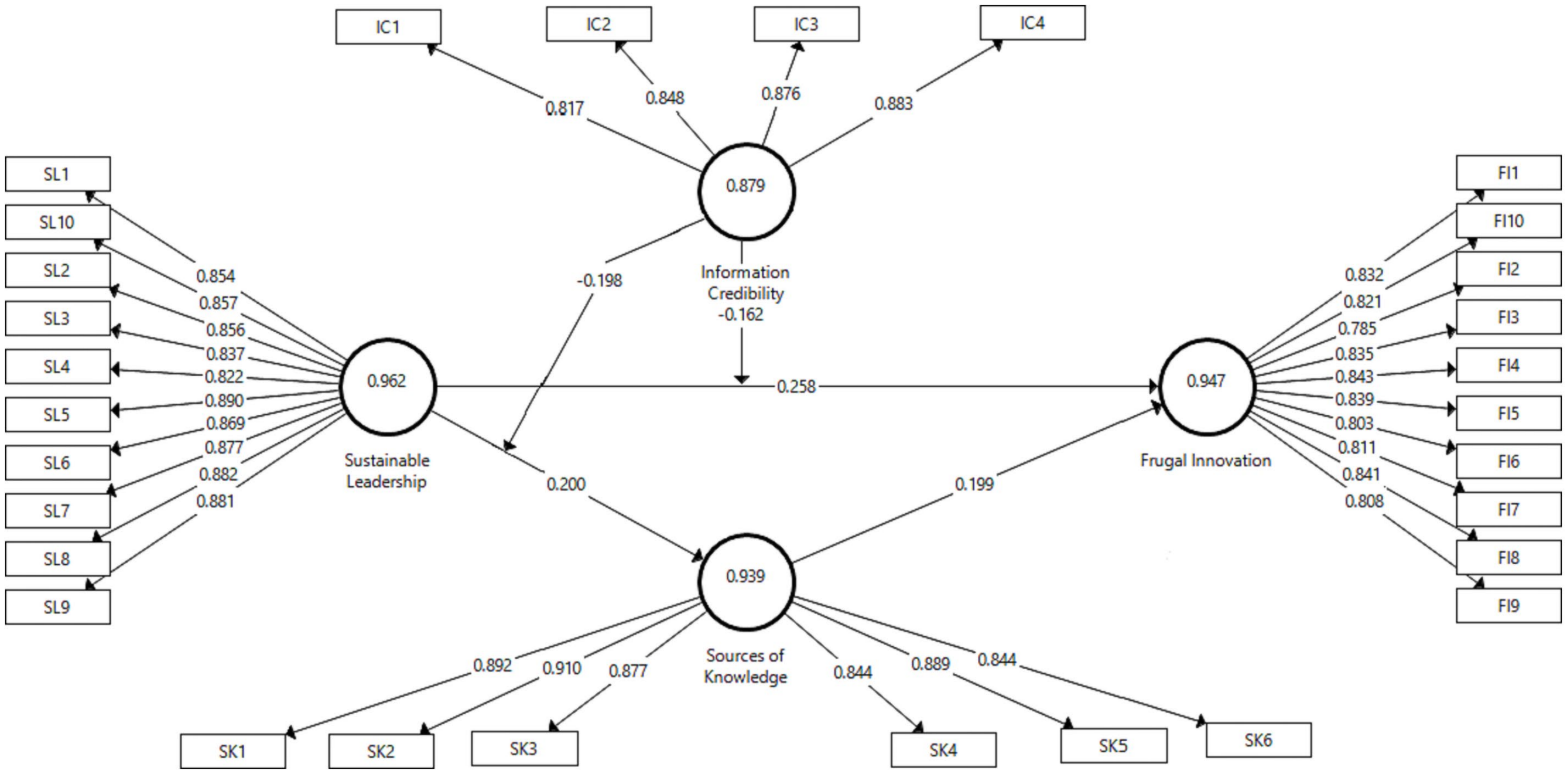
Structural model.

Findings from the Structural Model Path Analysis illuminate the interdependencies between the theoretical framework’s many moving parts (see Figure 2). Effective Leadership has a path coefficient of 0.218 with Economical Innovation. According to these results, Sustainable Leadership greatly benefits Frugal Innovation. There is a variance of 0.035 according to the standard deviation. The value of p is 0.000, while the T-statistic is 6.17. These statistics show that there is a statistically substantial link between Sustainable Leadership and Frugal Innovation.

A path coefficient of 0.200 shows the link between Sustainable Leadership and Sources of Knowledge. This shows that Sustainable Leadership affects Sources of Knowledge in a good way. The standard deviation is equal to 0.055. This experiment has a value of p of 0.000 and a T-statistic of 3.673. At the 0.05 level, the results show that there is a statistically significant link between Sustainable Leadership and Sources of Knowledge.

The path coefficient between Information Credibility * Sustainable Leadership (moderating variable) and Frugal Innovation is−0.162. This indicates that the interaction between Information Credibility and Sustainable Leadership has a significant negative effect on Frugal Innovation. 0.021 is the standard deviation. The value of p is 0.000, and the T-statistic is 7.849. This means that at the 0.05 level, there is a statistically significant relationship between the interaction of Information Credibility, Sustainable Leadership, and Frugal Innovation.

Using PLS-SEM, the current study looked at the path coefficients between Information Credibility * Sustainable Leadership (moderating variable) and Sources of Knowledge, and between Sources of Knowledge and Frugal Innovation. When looking at the correlation between Information Credibility and Sustainable Leadership, the value of−0.198 shows a significant negative effect on the Sources of Knowledge. Statistics were significant at the 0.05 level, as indicated by the T-statistic of 7.415, the standard deviation of 0.027, and the value of p of 0.000. The positive correlation between Sources of Knowledge and Frugal Innovation was measured by this coefficient, which was calculated as 0.199. The value of p was 0.000, the T-statistic was 4.683, and the standard deviation was 0.043, all of which pointed to statistical significance at the 0.05 level.

In general, the findings of the path analysis lend support to the assumptions that guided our research (see Table 7). The significant positive path coefficients support the first and second hypotheses, which say that Sustainable Leadership has a positive effect on Frugal Innovation and Sources of Knowledge, respectively. A substantial positive path coefficient supports the final hypothesis, which suggests that Sources of Information have a positive impact on Economical Creativity. Significant negative path coefficients support the third and fourth hypotheses, which are moderation hypotheses that claims that the interaction between information credibility and sustainable leadership moderates the link between sustainable leadership and frugal innovation, and sources of knowledge, respectively.

**Table 7:** Data coefficient for direct paths.

	Original sample	Standard deviation	T statistics	*P* values
Sustainable leadership - > Frugal innovation	0.218	0.035	6.170	**0.000**
Sustainable leadership - > Sources of knowledge	0.200	0.055	3.673	**0.000**
Information credibility * Sustainable leadership - > Frugal innovation	−0.162	0.021	7.849	**0.000**
Information credibility * Sustainable leadership - > Sources of knowledge	−0.198	0.027	7.415	**0.000**
Sources of knowledge - > Frugal innovation	0.199	0.043	4.683	**0.000**

### Mediating effect

The intermediary path analysis shows that sustainable leadership has a significant direct effect on frugal innovation (0.218, *p* = 0.001) and a significant indirect effect on frugal innovation (0.040, *p* = 0.011) through sources of knowledge. Based on these results, we can say that Sources of Knowledge act as a bridge between Sustainable Leadership and Frugal Innovation.

Moreover, the results of the moderation analysis demonstrate that the interaction between Information Credibility and Sustainable Leadership significantly weakens the indirect path from Sustainable Leadership to Frugal Innovation through Sources of Knowledge (−0.039, *p* < 0.001). As a result, the mitigating impact of Information Reliability moderates the relationship between Sustainable Leadership and Economical Creativity through Sources of Knowledge to a great extent.

Overall, these results suggest that Sustainable Leadership has a positive effect on Frugal Innovation, and that Sources of Knowledge can help explain why this is the case. But the connection between Sustainable Leadership, Sources of Knowledge, and Frugal Innovation depends on how credible the information is. The detailed findings are presented in Table 8.

**Table 8:** Data coefficient for mediating effects.

	Original sample	Standard deviation	*T* statistics	*P* values
Sustainable leadership - > Sources of knowledge - > Frugal innovation	0.040	0.016	2.535	**0.011**
Information credibility * Sustainable leadership - > Sources of knowledge - > Frugal innovation	−0.039	0.009	4.233	**0.000**

## Discussion

The goal of this study was to find out how sustainable leadership and knowledge sources affect frugal innovation in SMEs in Pakistan, as well as how the credibility of information affects these relationships. The study looked at 325 workers from SMEs in Pakistan that contribute to making goods or provide services. PLS-SEM was used to look at the data.

The *first hypothesis (H1)* of the research is supported by the results of this study, which show that sustainable leadership has a substantial favorable effect on frugal innovation. This result is consistent with previous research demonstrating a positive correlation between sustainable leadership and innovation in organizations (Burawat, 2019). Sustainable leadership is characterized by a long-term vision, social responsibility, and ethical behavior, which are all conducive to promoting innovation in organizations. The *second* hypothesis (*H2*), which proposed that sustainable leadership significantly impacts the sources of knowledge, was also supported by the results. Sustainable leadership was found to have a beneficial and substantial effect on the use of knowledge sources by Pakistani SMEs and SMBs. This result is in line with what other studies have found, which is that leaders can play a big role in getting their companies to share and create new knowledge (Iqbal and Piwowar-Sulej, 2023).

The study also found that the relationship between sustainable leadership and frugal innovation is significantly changed by the credibility of the information. This supports Hypothesis *H3*. This result indicates that when information credibility is strong, the effect of sustainable leadership on cost-effective or frugal innovation is magnified. This is because credible information helps employees to better understand the vision and goals of the organization and align their behavior accordingly, which can lead to greater innovation. This finding is in line with the previous research (Khattak et al., 2022). The results also provided evidence for Hypothesis *H4*, which suggested that information credibility moderates the association between sustainable leadership and knowledge sources. This suggests that the impact of sustainable leadership on knowledge sources is more pronounced when information credibility is high. This finding highlights the importance of credible information in promoting knowledge sharing and creation in organizations; also these findings get support from the previous research (Lei et al, 2021).

The results back up the *fifth hypothesis (H5)*, which said that sources of knowledge have a big effect on frugal innovation. This finding shows that it’s important for SMEs to have access to a wide range of reliable sources of knowledge in order to encourage frugal innovation. This is in line with earlier studies that found that companies’ access to information can foster innovation (Broadbent et al., 2019). The results also confirmed the *sixth hypothesis (H6)*, which suggested that knowledge sources influence the link between sustainable leadership and frugal innovation. This suggests that knowledge sources play a role in the positive effect that sustainable leadership has on frugal innovation. This finding shows how important knowledge sources are in making it easier for sustainable leadership to have an effect on innovation. This result is consistent with previous research (Lei et al., 2021).

The examination of the relationships between sustainable leadership, sources of knowledge, information credibility, and frugal innovation within Small and Medium-sized Enterprises (SMEs) provides a nuanced understanding of the intricate dynamics at play. The results suggest that sustainable leadership serves as a significant driver, positively influencing both frugal innovation and the acquisition of knowledge sources. This finding underscores the importance of leadership practices that incorporate economic, environmental, and social considerations, fostering innovation and knowledge accumulation in SMEs. Furthermore, the interaction between sustainable leadership and information credibility introduces a layer of complexity to the relationships. The negative impact observed suggests that the credibility of information can significantly shape the effectiveness of sustainable leadership in influencing frugal innovation and sources of knowledge. This emphasizes the need for leaders to not only embody sustainable principles but also ensure the credibility of information guiding their decision-making processes. Exploring the mediating effects, the study reveals that sources of knowledge play a crucial role in translating the influence of sustainable leadership into frugal innovation. This indirect pathway highlights the importance of knowledge acquisition and dissemination mechanisms within SMEs as a key mediator for sustainable leadership’s impact on innovation outcomes.

In the end, this study showed that information credibility has a big effect on the relationship between sustainable leadership and sources of knowledge, which act as a bridge between sustainable leadership and frugal innovation in Pakistani SMEs (*H7*). Based on these results, it seems that the good effects of sustainable leadership on knowledge sources and frugal innovation are stronger when the credibility of information is high. Previous findings were also in line with this research findings (Dost et al., 2019). *Overall*, this study provides valuable empirical evidence on the relationship between sustainable leadership, knowledge sources, and frugal innovation in SMEs in Pakistan. Additionally, the study underscores the important moderating role of information credibility in these relationships. The implications of these findings for managers and leaders in SMEs are noteworthy, as they can leverage this knowledge to develop effective strategies for fostering innovation in their organizations. Future research can build upon these findings by exploring additional moderators or mediators of the relationships under investigation.

## Conclusion

The goal of this study was to find out how sustainable leadership and knowledge sources affect frugal innovation in SMEs, as well as how the credibility of information affects these matters. Statistical evidence supported the acceptance of all hypotheses. This suggests that sustainable leadership and knowledge sources are important for driving frugal innovation in SMEs in Pakistan, and that the credibility of information has a big effect on the relationship between these variables. The results of this study are important for SMEs in Pakistan in a number of ways. *Firstly*, sustainable leadership is crucial for promoting frugal innovation in these firms. Leaders who prioritize sustainability and are committed to driving innovation through efficient resource use can create a culture of innovation within their organizations. This can help SMEs to stay competitive in a rapidly changing business environment. *Secondly*, the study highlights the importance of knowledge sources in driving frugal innovation. The findings suggest that SMEs should strive to create a knowledge-sharing culture within their organizations, which can help them to access new ideas and best practices. This can be achieved through formal training programs, mentoring, and collaboration with external partners. *Thirdly*, credibility of information was found to moderate the link between sustainable leadership and frugal innovation. This means that SMEs should try to set up ways to check information and make sure it is correct and trustworthy. This can help build trust and credibility within the organization, which can be important for driving innovation. Overall, the study provides valuable insights into the factors that can drive frugal innovation in SMEs in Pakistan. The results suggest that innovation is driven by strong and sustainable leadership and sources of knowledge, and that the credibility of information has a big effect on this relationship. The results of this study can be used by SMEs to come up with plans for promoting cheap and frugal innovation and staying competitive in an ever-changing business world.

## Implications

This research yields several significant implications based on its findings. These implications are delineated into two distinct subsections: theoretical and practical.

### Theoretical implications

This study enlightens prospective business leaders in Pakistan on the topics of sustainable leadership, knowledge sources, information credibility, and cost-effective or frugal innovation. *To begin*, the study contributes to the existing literature on frugal innovation by giving empirical evidence for the positive impacts of sustainable leadership on frugal innovation among small and medium-sized businesses. The results of this study show that SMEs, especially in developing countries like Pakistan, need stable and sustainable leadership to be able to innovate on a budget. *Second*, the research shows how important sources of knowledge are for small and medium-sized businesses that want to innovate on a budget. The results show how important it is for small and medium-sized businesses (SMBs) to make and get access to a wide range of knowledge sources in order to support frugal innovation. This result shows that small and medium-sized businesses should use open innovation practises to get access to a wide range of sources of knowledge. It also has important theoretical implications for innovation management. *Lastly*, the research shows how the credibility of information plays a role in the link between sustainable leadership, sources of knowledge, and frugal innovation at SMEs. The results show that the credibility of information is a key factor in figuring out how effective sustainable leadership and knowledge sources are at getting businesses to be more creative with their money. This result adds to the research on the credibility of information and shows that small and medium-sized businesses should prioritize improving the reliability of their knowledge sources to encourage frugal innovation.

### Practical implications

The findings of this study also have important managerial implications for SMEs in Pakistan. *Firstly*, SMEs should invest in sustainable leadership practices to promote frugal innovation. The study suggests that sustainable leadership practices such as promoting employee participation, empowering employees, and promoting ethical and social responsibility can enhance frugal innovation in SMEs. *Secondly*, SMEs should focus on developing and accessing diverse knowledge sources to promote frugal innovation. The study suggests that SMEs can benefit from open innovation practices and collaboration with external partners to access diverse knowledge sources. *Thirdly*, SMEs should focus on enhancing the credibility of their knowledge sources to promote frugal innovation. The study suggests that SMEs should ensure that their knowledge sources are reliable, accurate, and trustworthy to enhance their effectiveness in driving frugal innovation. In *conclusion*, the findings of this study highlight the importance of sustainable leadership, sources of knowledge, and information credibility in promoting frugal innovation in SMEs in Pakistan. The study gives additional knowledge about what makes frugal innovation happen and why. It also has important managerial and theoretical implications for small and medium-sized enterprises (SMEs) in Pakistan and other emerging economies.

## Limitations and future directions

Despite its merits, this research has some flaws that should be taken into account when interpreting the findings about the association between sustainable leadership, knowledge sources, information credibility, and cost-effective or frugal innovation. *First* of all, the study was only done on SMEs in Pakistan. Because cultural and economic factors could change the results, the results cannot be generalized to other areas of the economy or countries as a whole. Therefore, further research is required to confirm the validity of the findings in other contexts. *Secondly*, this study used a cross-sectional design. Although the data collection was conducted at two different times, it is not possible to infer causality as a longitudinal design would allow. Future research could adopt a longitudinal design to explore causal relationships between sustainable leadership, knowledge sources, information credibility, and frugal innovation. *Thirdly*, the study collected data from employees of SMEs involved in the production of services or goods. It is possible that the results may differ for SMEs involved in other industries, such as finance or technology. Future research should explore the relationship between sustainable leadership, knowledge sources, information credibility, and frugal innovation in different industries. *Fourthly*, the sample size may not represent full population, but in future this can be eliminated by using more data. *Finally*, this study used self-reported measures, which may have introduced response bias. Future research could adopt other measures, such as objective performance measures, to reduce the potential for response bias.

There are several directions for future research suggested by this study. *Firstly*, researchers could explore the role of additional variables, such as moderating role of organizational culture or employee engagement, in the association between sustainable leadership, knowledge sources, information credibility, and frugal innovation. *Second*, since this study found that knowledge sources are a link between sustainable leadership and frugal innovation, future studies might look at other possible links, such as mediating roles of organizational effectiveness or teamwork, to learn more about this relationship. *Third*, this study showed that the relationship between sustainable leadership and frugal innovation is moderated by how credible the information is. Researchers could look into other possible moderators, like the size of the organization or the competition in the industry, to find out what might make this relationship stronger or weaker. SMEs in Pakistan were also looked at in this study. Future research could look at the relationship between sustainable leadership, knowledge sources, the information credibility, and frugal innovation in other countries or regions to see if the results are the same in all of them.

## Data availability statement

The raw data supporting the conclusions of this article will be made available by the authors, without undue reservation.

## Author contributions

KU: Writing – review & editing, Data curation. RA: Writing – original draft, Writing – review & editing. VA: Resources, Validation, Writing – review & editing. UA: Conceptualization, Writing – original draft. CF: Conceptualization, Software, Validation, Writing – review & editing. ML: Conceptualization, Methodology, Validation, Writing – review & editing.
