# Supplementary material for: Driving frugal innovation in SMEs: how sustainable leadership, knowledge sources and information credibility make a difference
Source: Front Sociol. 2024 Mar 1;9:1344704. doi: 10.3389/fsoc.2024.1344704 (PMC10941759; doi:10.3389/fsoc.2024.1344704)
Supplement: Supplementary file 1 [file Data_Sheet_1.docx]

**Section 1: Demographic Information**

1. What is your age?
2. What is your gender?
3. How many years of experience do you have in your current SME?
4. What is your current role within the SME?
5. What is the size of the SME you work for?

**Frugal innovation** (Yousaf et al., 2022)

Evaluate your SME's approach towards frugal innovation based on the following statements:

1. How frequently does the SME focus on core functionality over additional features?
2. To what extent does the SME seek novel solutions for improvement?
3. How much attention does the SME pay to enhancing the durability of its services or products?
4. How consistently does the SME offer good-quality, cost-efficient services/products?
5. How actively does the SME work on reducing production costs?
6. How often does the SME aim to decrease the final price of products/services?
7. To what extent does the SME prioritize environmental sustainability in its operations?
8. How involved is the SME in partnerships with local businesses?
9. How effectively does the SME find innovative ways to meet environmental needs?
10. How successful is the SME in meeting customer needs by offering cost-efficient products?

**Sustainable Leadership** (Dalati, Raudeliuniene, & Davidavičienė, 2017)

Please rate the following statements based on your observations of leadership within your SME:

1. How often does the leadership show a strong comprehension and attentive listening to employees' opinions?
2. How frequently does the leadership consider and value the diverse backgrounds and values of team members?
3. To what extent does the leadership keep all communication channels open and inform the team about decisions made?
4. How aware is the leadership of organizational factors that could hinder the SME's goals?
5. How often does the leadership collaborate with others and interpret sector trends to drive the organization forward?
6. How trustworthy and reliable is the leadership in keeping promises and earning trust?
7. How effectively does the leadership set future-oriented tasks and goals for the organization?
8. To what extent does the leadership work towards establishing a collective team identity?

**Sources of Knowledge** (Broadbent, Metternicht, & Drozdzewski, 2019)

Please indicate the extent to which you rely on the following sources for acquiring knowledge related to your work in the SME:

1. Prior knowledge about specific areas relevant to your work.
2. Input and information from family and friends.
3. Information obtained from car salespersons or relevant industry sales representatives.
4. Formal information sources (e.g., magazine articles, websites, articles by industry experts, TV shows).
5. Advertisements about specific products or services.
6. Personal experience through test driving or trying out new methods/products.

**Information credibility** (Li & Suh, 2015)

Regarding the credibility of information sources, please rate the following statements:

1. How believable do you find the information presented on certain platforms (e.g., social media, company websites)?
2. To what extent do you believe the information provided on these platforms is factual?
3. How credible do you perceive the information shared on these platforms?
4. How trustworthy do you consider the information shared on these platforms?

**Knowledge acquisition** (Guo, Wang, Wang, & Zhang, 2019)

***Technical knowledge acquisition***

1. Our firm acquires technology through licensing.
2. Our firm acquires technology through R&D contracting.
3. Our firm acquires technology through take-over.
4. Our firm acquires technology through hiring away personnel.

***Market knowledge acquisition***

1. Our firm has processes for continuously collecting information from customers.
2. Our firm has processes for continuously collecting information about competitor activities.
3. Our firm has processes for continuously collecting information from our suppliers (MKA3).

References

Broadbent, G., Metternicht, G., & Drozdzewski, D. (2019). An Analysis of Consumer Incentives in Support of Electric Vehicle Uptake: An Australian Case Study. *World Electric Vehicle Journal, 10*, 11. doi: 10.3390/wevj10010011

Dalati, S., Raudeliuniene, J., & Davidavičienė, V. (2017). Sustainable Leadership, Organizational Trust on Job Satisfaction: Empirical Evidence from Higher Education Institutions in Syria. *Business, Management and education, 15*. doi: 10.3846/bme.2017.360

Guo, Y., Wang, L., Wang, M., & Zhang, X. (2019). The mediating role of environmental innovation on knowledge acquisition and corporate performance relationship—A study of SMEs in China. *Sustainability, 11*(8), 2315.

Li, R., & Suh, A. (2015). Factors Influencing Information credibility on Social Media Platforms: Evidence from Facebook Pages. *Procedia Computer Science, 72*, 314-328. doi: https://doi.org/10.1016/j.procs.2015.12.146

Yousaf, Z., Panait, M., Tanveer, U., Cretu, A., Hrebenciuc, A., & Zahid, S. M. (2022). Value Creation through Frugal Innovation, Innovation Capability and Knowledge Sharing in a Circular Economy. *14*(14), 8504.
